# Protein Function Assignment through Mining Cross-Species Protein-Protein Interactions

**DOI:** 10.1371/journal.pone.0001562

**Published:** 2008-02-06

**Authors:** Xue-wen Chen, Mei Liu, Robert Ward

**Affiliations:** 1 Bioinformatics and Computational Life-Sciences Laboratory, Information and Telecommunication Technology Center (ITTC), Department of Electrical Engineering and Computer Science, The University of Kansas, Lawrence, Kansas, United States of America; 2 Department of Molecular Biosciences, The University of Kansas, Lawrence, Kansas, United States of America; University of Dayton, United States of America

## Abstract

**Background:**

As we move into the post genome-sequencing era, an immediate challenge is how to make best use of the large amount of high-throughput experimental data to assign functions to currently uncharacterized proteins. We here describe CSIDOP, a new method for protein function assignment based on shared interacting domain patterns extracted from cross-species protein-protein interaction data.

**Methodology/Principal Findings:**

The proposed method is assessed both biologically and statistically over the genome of *H. sapiens*. The CSIDOP method is capable of making protein function prediction with accuracy of 95.42% using 2,972 gene ontology (GO) functional categories. In addition, we are able to assign novel functional annotations for 181 previously uncharacterized proteins in *H. sapiens*. Furthermore, we demonstrate that for proteins that are characterized by GO, the CSIDOP may predict extra functions. This is attractive as a protein normally executes a variety of functions in different processes and its current GO annotation may be incomplete.

**Conclusions/Significance:**

It can be shown through experimental results that the CSIDOP method is reliable and practical in use. The method will continue to improve as more high quality interaction data becomes available and is readily scalable to a genome-wide application.

## Introduction

Genome sequencing projects have deposited tremendous amounts of protein sequence data for a vast number of genomes, and as we move into the post-genomic era, it will be crucial to determine biological functions for all these encoded proteins. Currently, a substantial portion of most genomes is still unannotated [1]. For instance, among the current list of *Drosophila* genes downloaded from FlyBase (November 2006) [2], only 54% are annotated with “molecular function” terms in gene ontology (GO) [3]. Additionally, many proteins are modular, consisting of multiple functional domains, and therefore the existing annotations may still be incomplete.

While experimental methods such as loss of function mutational analysis, RNAi, or targeted misexpression approaches have been very successful in identifying protein functions, they are labor intensive and time consuming. As a result, much of the genome-wide functional annotations are based upon *in silico* methods. The most established computational approaches to function detection primarily depend on homology matching to genes with known functions utilizing programs such as FASTA [4] and PSI-BLAST [5]. However, assuming functional annotations by sequence similarity poses some critical questions, such as at what level of sequence similarity can we feel assured that the two proteins carry out the same function, and at what level of detail if the function is conserved? Over the years, numerous non-homology based computational techniques have been developed to derive protein functions from additional sources of biological data such as gene fusion events [6], [7], phylogenetic profiles of proteins in multiple genomes [8], gene expression and mutant phenotype data [9], and heterogeneous data such as gene expression, physical interactions, motif information and transcription factor binding sites data [10]–[13].

With the ever-increasing accumulation of high-throughput protein-protein interaction data, a number of computational approaches have emerged to take advantage of these data for gene function prediction [14]–[21]. In general, these approaches are based upon the premise that proteins often physically interact to achieve a common objective. Hence, it may be possible to infer functions for a protein based on its interaction partners. The concept is also known as ‘guilt-by-association’, which assumes that interacting proteins are more likely to carry out similar functions. Schwikowski et al. [14] applied a neighbor counting method where unknown proteins were assigned functions based on the frequencies of their interaction partners having particular functions. Thereafter, several research groups attempted to improve the neighbor counting method through application of χ2 statistics [15], Bayesian analysis [16], and Markov random field analysis [17], [18]. Moreover, several researchers have introduced protein interaction network based methods [19], [20], and Brun et al. [21]–[24] clustered the Saccaromyces cerevisiae proteome into several groups to predict cellular functions using protein interaction data.

Although most computational methods have shown great promise in function assignment, current methods still suffer from two major limitations. First, most function prediction algorithms can predict protein functions with 50%–75% accuracy, which may not be of practical use for biologists. Moreover, some methods use only several tens to hundreds of functional categories in the prediction process which resulted in more generic rather than specific functional assignments. Therefore, developing more effective *in silico* methods to increase the fidelity of these functional annotations and to propose novel functions for currently uncharacterized proteins presents a major challenge to the life science community and will eminently aid the biological community as higher quality functional annotations are often used by scientists to generate new hypotheses and direct their research focus.

In this paper, we describe CSIDOP, Cross-Species Interacting DOmain Patterns, a new method for protein function assignment based on the shared interacting domain patterns extracted from cross-species protein-protein interaction data. In an evaluation of the CSIDOP method we use protein-protein interaction data from the *Homo sapiens* genome, and find that CSIDOP is capable of making molecular function predictions for human proteins with accuracy of 95.42% using 2,972 gene ontology (GO) functional categories (the most specific terms in GO). In addition, CSIDOP is able to assign novel functional annotations for 181 previously uncharacterized proteins. Furthermore, we demonstrate that CSIDOP can complement current GO annotation by providing additional functional annotations for proteins that are already characterized by GO.

## Results

### Principle of the CSIDOP method

The CSIDOP method tackled the protein function determination problem by analyzing interacting domain patterns that are conserved across different species. A brief synopsis of the method is presented here with a more detailed description presented in “Materials and methods”. Protein domains are the structural and/or functional units of proteins. They are conserved through evolution and serve as the building blocks of proteins. Some protein domains serve specific functions such as tyrosine kinase domains that covalently attach phosphate groups to select tyrosine residues in target proteins, whereas other protein domains may be more generic, for example participating in protein-protein binding and thereby being associated with numerous biological activities. A protein may contain only a single domain or it may contain multiple domains. In some cases multiple domains may work together for the execution of a single function. Protein functions are often directed by physical interactions of these modular domains [25]. Pereira-Leal and Teichmann [26] suggested that protein interactions often evolve through duplication of the proteins involved in the interaction. In their work, partial duplicates are defined as any two interaction pairs with one protein in common and homology between the other proteins. Any two interactions where both proteins are homologous are counted as complete duplicates. Their results indicated that the duplicated modules tend to retain similar general functions. This suggests that interacting modular domains may be conserved over time and between organisms. Moreover, a shared pattern between two interacting protein pairs may indicate that both pairs interact through the same shared modular domains. We are exploring this property of conservation of interaction as a means to assign protein functions by concentrating on protein-protein interaction (PPI) pairs with similar interacting modular domain patterns.

**Figure 1:**
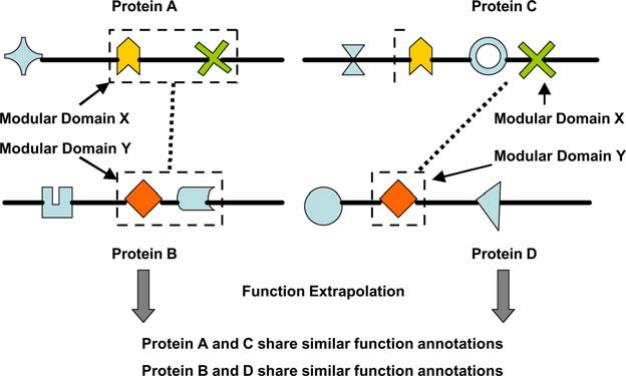
Function annotation scheme based on interacting domain patterns. This also illustrates how domain interaction can contribute to protein interactions. One or more domains in a protein may form modular domains and interact with other modular domains in other proteins. Dashed rectangles represent modules. In each module, one or more domains may exist and form a unit during interaction. The dashed lines represent interactions between proteins. Since the protein-protein interaction pairs A–B and C–D share common domain interaction patterns, and proteins A and C and B and D share the same interacting modular domains, we may deduce that the proteins are associated with similar functional annotations.

Under this hypothesis, if two PPI pairs contain a common interacting domain pattern, then proteins in the two pairs with similar modular domains are more likely to be associated with similar functions. For example, assume that there exist two PPI pairs: protein A interacts with protein B and protein C interacts with protein D. If proteins A and C contain the same modular domain X that interact with the modular domain Y in proteins B and D, then we conclude that the two PPI pairs share a common interaction domain pattern. Therefore, we extrapolate that proteins A and C are more likely to have similar functions, and the same applies to proteins B and D (Figure 1).

We first explored this concept for PPI pairs from different species and have observed evidence of this conservation of function between the PPI pairs. For example, in *C. elegans*, nhr-67 [Swiss-Prot: Q9XVV3] and daf-21 [Swiss-Prot: Q18688] have been shown to interact [27], whereas in human ESR1 [Swiss-Prot: P03372] and HSP90AA1 [Swiss-Prot: P07900] are also known to interact [28]. Both PPI pairs contain a common domain interaction pattern, (PF00105)-(PF02518, PF00183), where ‘-’ denotes interaction and the parentheses denote modular domains. PF00105 is described by Pfam [29] as the zinc finger, C4 type domain, and PF02518 and PF00183 refer to HATPase_c and HSP90 domains, respectively. The proteins nhr_67 and ESR1 contain the PF00105 domain, whereas daf-21 and HSP90AA1 contain the modular domain (PF02518, PF00183). In the Gene Ontology database [3], the proteins nhr-67 in *C. elegans* and ESR1 in human are annotated to the same function terms such as ligand dependent nuclear receptor activity, regulation of transcription, DNA dependent, DNA binding, and transcription factor activity. Analogously, daf-21 and HSP90AA1 were found to be annotated with the same function terms, ATP binding and protein folding.

It is important to note that this method is fundamentally different from other protein interaction-based function detection algorithms where the function of a target protein is determined strictly by its interaction partners. Compared with existing methods, our method is distinctive in the following aspects: (i) protein functions are detected through the shared interacting domain patterns, (ii) the patterns are mined from the cross-species protein interaction data, and (iii) unknown proteins can be assigned to various functional categories in GO, in contrast to most other methods where proteins are assigned with a limited number of functional categories such as MIPS [30] that are less specific than GO. A complete description of the experimental results, novel protein function discoveries, and design of our model are given in the sections below.

### Biological and statistical evaluation of the CSIDOP on H. sapiens

An essential issue concerning the protein function prediction problem is the assessment of method reliability. To evaluate the CSIDOP method, a set of protein interaction data is partitioned into two groups: (1) training data: PPI pairs where both proteins are annotated in the GO, and (2) testing data: PPI pairs with at most one protein annotated. The training dataset is used to extract interacting domain patterns. The test dataset, on the other hand, contains interaction pairs that have either one of the proteins uncharacterized or both unknown. Thus, we can assess the reliability of the CSIDOP method by determining how well it worked in function prediction for those GO-characterized proteins and predict functions for proteins that are currently not characterized in GO in the test dataset.

We chose to evaluate the method using proteins in *H. sapiens*. The collected human protein interaction data were separated exclusively into training and test datasets as described above. To train the CSIDOP method, we integrated protein-protein interaction (PPI) data from the organisms *S. cerevisiae*, *C. elegans*, and *D. melanogaster*, in addition to the large data set from *H. sapiens*. In order to assess the relative performance of our method, inferred functions of the *H. sapiens* proteins (by CSIDOP) were then compared to the known functions in the GO database, which we designate as the ‘true’ terms. Hence throughout this paper, the ‘true’ function terms of a protein refer to the known function terms of this protein listed in the GO. An exact match between a predicted term and the corresponding true GO term for a protein indicates a correct prediction; and wrong prediction otherwise.

### Comparison of the CSIDOP method with other methods

After training, CSIDOP produced a lookup table of significant interacting modular domain patterns from interaction pairs in the training dataset (see Text S1 and Text S2), where each pattern is associated with a number of function terms (please refer to “Material and Method” for details). Annotations were made to a PPI pair in the test dataset if it contains at least one interacting modular domain pattern listed in the table. Overall, we could assign functions for 618 *H. sapiens* proteins from PPIs with common domain patterns in the lookup table. Among the 618 predicted proteins, 437 had existing annotations in the GO database, and thus could be used to evaluate the CSIDOP method. Among the 437 proteins, 417 were assigned with correct functions by the CSIDOP (assigned functions have an exact match with the ‘true’ terms), i.e., the CSIDOP method had an accuracy of 95.42% (Table 1) using 2,972 GO functional terms, which is higher than most of the existing *in silico* methods. For comparison, we also tested the Majority Rule (MR) method by Schwikowski et al. [14], a simple domain based method, and orthology based method.

**Table 1:** Method Comparison

Method	Accuracy
CSIDOP	95.42%
Majority Rule (MR)	59.50%
Pfam domain based method	61.98%
Orthology based method	83.86%

Accuracy of the CSIDOP, Majority Rule (MR), Pfam domain based, and orthology based methods are compared in protein function prediction. The accuracy is defined as the percentage of proteins predicted with correct function terms. A protein is considered to be correctly annotated if the known function occurred among the predicted terms.

Generally, the MR algorithm assigns a protein with the most frequent function terms among its direct interaction partners. Assessing the MR algorithm on the same target dataset that we used in CSIDOP, MR made functional predictions with an accuracy of 59.50% (Table 1). As for the domain based method, considering the fact that a number of protein domains are annotated in Pfam [29] with specific functions, and thus it is possible to make protein function predictions according to the functional terms associated with its domains. Using the same set of proteins, only 61.98% were assigned with correct functions using the simple domain based scheme (Table 1). Lastly, for the orthology based method, we attempted to assign functions to proteins according to their annotated orthologs in other species. The orthologs were retrieved using Inparanoid [31]. The orthology based method achieved prediction accuracy of 83.86%, and among the novel predictions, it only covered 56.35% of our novel discoveries. Therefore, our CSIDOP method can provide an extra power in protein function prediction compared to the orthology detection.

Most existing methods have been evaluated on the *S. cerevisae* proteome using a smaller number of functional categories. Schwikowski et al. [14], Hishigaki et al [15], and Brun et al [21] used 42, 41, and 44 “cellular role” categories in the Yeast Protein Database (YPD) [32], and the accuracies achieved were 72%, 64%, and 67%, respectively. In [19], Vazquez et al. evaluated their method using two different level of functional classification in MIPS [30]. In the coarse-grained level containing only 20 functional categories, the accuracy was about 83%. In the finest level containing 424 functional categories, the accuracy decreased to 65%. Noticeably, the CSIDOP prediction was made over 2,972 GO functional categories, which is significantly larger than those employed in other methods. Accordingly, the assigned functions were specific rather than generic. In principle, the more coarse-grained the classification, the easier the prediction is. Applying the same definition of success, our CSIDOP method was able to make correct predictions an astounding 95.42% of the time using the full 2,972 GO molecular function categories. However, in the GO function tree, the closer a node is to the root, the lower the level in GO tree, which means that the corresponding function is more abstract and the farther it is from the root, the higher the level in GO tree, thus the more detailed. An important advantage of the CSIDOP method is that it can be tailored to different levels in the GO database based upon need. For example, suppose that GO level is set to five, then all predicted terms at GO tree levels higher than or equal to five will be generalized to the corresponding function at level five. In other words, the more specific functional terms that reside at higher levels of the tree are replaced with their ancestor terms which are located at level five. Higher prediction accuracy is expected as we lower the GO depth. Consistent with this, the prediction accuracy in the test dataset reached 98.85% when the depth parameter was set to 2, which still contains 129 GO functional categories (Table 2). Table 2 shows the prediction accuracy as a function of GO level for this test dataset and indicates the robustness and reliability of the CSIDOP method. This depth parameter allows users to assign function terms for a protein at different resolutions according to their individual needs.

**Table 2:** Evaluation of the CSIDOP algorithm.

Depth in the GO graph	# of unique GO functional categories	# of correctly Predicted proteins	# of predicted proteins different from their GO terms	Prediction accuracy
2	129	432	5	98.85%
3	473	427	10	97.71%
4	961	422	15	96.56%
5	1996	419	18	95.88%
6	2598	418	19	95.65%
7	2816	417	20	95.42%
8	2938	417	20	95.42%
9	2957	417	20	95.42%
10	2972	417	20	95.42%

Accuracy is assessed over a number of values for the depth parameter (i.e. generalizing annotated terms when parameter decreases). A protein is considered to be correctly annotated if the known function occurred among the predicted terms.

### CSIDOP complements the current GO annotation

A protein often exhibits multiple molecular functions and its annotation in GO may therefore not be complete. For the 20 proteins with predicted functions that do not match with their ‘true’ terms, the differences between the predicted terms and the ‘true’ terms may be due to incompleteness of the GO annotations. Moreover, the CSIDOP may provide additional functional terms to existing proteins. For example, the Alpha-2-macroglobulin precursor [Swiss-Prot: P01023], was predicted by CSIDOP to be involved in protease inhibitor activity (GO:0030414), which is not among the current list of functions annotated in GO. Consistent with this prediction, alpha-2-macroglobulin is found to be a major human plasma protease inhibitor capable of inhibiting most endopeptidases tested [33]. Another example is the PRS7 [Swiss-Prot: P35998] gene in human, which is currently annotated in GO to participate in protein binding (GO:0005515), with no other listed terms. Our CSIDOP method predicted that it is also involved in ATP binding (GO:0005524), hydrolase activity (GO:0016787), nucleotide binding (GO:0000166), and nucleoside_triphosphatase activity (GO:0017111), all of which can be verified in InterPro [34]. Other assigned terms for PRS7 by CSIDOP included endopeptidase activity (GO:0004175) and ATPase activity (GO:0016887), which were observed in the orthologous proteins of PRS7. An orthologous protein *in D. melanogaster*, RPT1 [Fly-Base: FBgn0028687], is annotated with endopeptidase activity inferred from direct assay [35]. Another orthologous protein in *S. cerevisiae*, YKL145W is also annotated with the function terms endopeptidase activity and ATPase activity.

**Figure 2:**
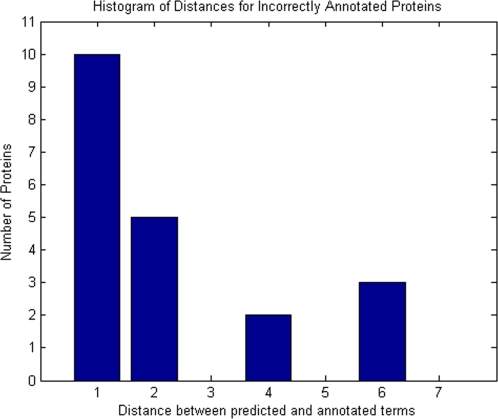
Histogram of distances between the wrongly predicted GO terms and the ‘true’ GO terms.

To gain insight into the 20 proteins that were “incorrectly” annotated by CSIDOP, we analyzed the relationship between the predicted terms and their true GO terms. Figure 2 shows a histogram of distances between the predicted terms and the ‘true’ GO terms, which is defined as the number of edges between these two terms in the GO graph. As illustrated in Figure 2, 15 out of the 20 proteins were predicted with function distances of one or two. A distance of one means that the two terms have a direct parent-child relationship; for instance, protein binding (GO:0005515) is a known function of Furin precursor protein [Swiss-Prot: P09958], and our method predicted it to be involved in protein domain specific binding (GO:0019904), which is a direct child term of protein binding in GO. If we consider such cases to also be successful prediction, then the accuracy improves from 95.42% to 97.71%. A distance of two indicates that the two terms share a parent. For example, suppressor of cytokine signaling 1 [Swiss-Prot: O15524] was identified in GO to be associated with insulin-like growth factor receptor binding (GO:0005159), whereas we assigned the function term, sevenless binding (GO:0005118). The two terms share a parent term, receptor binding (GO:0005102). In this case, if the more general terms were used, a correct functional annotation would have been achieved.

Moreover, we analyzed correlations between the predicted function terms and the ‘true’ terms. In GO, gene products can be associated with more than one term. Therefore, the correlation between two GO terms is defined based on the number of gene products in common [36]. The larger the correlation value is, the closer the two GO terms are. In order to assess the significance of the correlation scores between the predicted and ‘true’ terms, 10,000 GO term pairs were randomly selected, and a correlation score was computed for each pair. E-value is described as the probability of random GO term pairs achieving at least a certain correlation score. For instance, an E-value of 0.0008 implies that only eight out of the 10,000 random GO term pairs have scores equal to or higher than a particular correlation score. As a result, it was observed that among the 20 “incorrectly” annotated proteins, many predicted terms are closely correlated to the true GO terms with significant E-values. Table 3 shows the number of proteins versus different E-values. Examples of proteins in which extremely high correlation exists between the predicted and ‘true’ terms (E-value≤0.0008) are illustrated in Table 4.

**Table 3:** Correlation analysis for proteins with known terms that differ from predicted ones

E-value	Correlation Score (≥score)	# of Proteins
0.0116	1	19
0.0028	10	16
0.0021	20	14
0.0014	50	12
0.0008	100	10
0.0006	200	8
0.0005	300	6
0.0003	500	5
0.0001	3000	3
0.0000	10000	1

Correlation score between two GO terms is defined as the number of gene products in common. E-value is defined as the probability of random GO term pairs achieving at least a certain correlation score. The third column shows number of the wrongly predicted proteins reaching different correlation scores between predicted and ‘true’ terms.

**Table 4:** Examples of proteins with high correlation scores

Protein	True GO Term	Predicted GO Term	Direct Correlation Score
Partitioning defective 6 homolog alpha [Swiss-Prot: Q9NPB6]	GO:0017048 Rho GTPase binding	GO:0003779 Actin binding	186
SH3-containing GRB2-like protein 2 [Swiss-Prot: Q99962]	GO:0016740 Transferase activity	GO:0005509 Calcium ion binding	425
Hepatocyte growth factor precursor [Swiss-Prot: P14210]	GO:0004252 Serine-type endopeptidase activity	GO:0008233 Peptidase activity	6430
Erythrocyte membrane protein band 4.2 [Swiss-Prot: P16452]	GO:0005524 ATP binding	GO:0016740 Transferase activity	33762

Examples of proteins with predicted terms different from their ‘true’ terms but sharing high correlation scores (i.e. E-value≤0.0008). True GO term is the annotated term for a protein in GO. Predicted GO term is by the CSIDOP method.

### Novel function assignment for currently uncharacterized human proteins

Importantly, the CSIDOP predicted functional annotations for 181 *H. sapiens* proteins that are not currently described in the GO database. Some of these novel annotations can be supported with evidence provided by QuickGO, a web browser of gene ontology data maintained by the European Bioinformatics Institute [37]. For instance, the gene FHL1, four and a half LIM domains protein [Swiss-Prot: Q13642], was identified by the CSIDOP to participate in metal ion binding (GO:0046872) and zinc ion binding (GO:0008270). The metal ion binding annotation was found in QuickGO which was inferred from UniProt keywords. The zinc ion binding term was found by both the UniProt keywords and in InterPro [34], which is a database of protein families, domains and functional sites in which identifiable features found in known proteins can be applied to unknown protein sequences. Many novel functional annotations are supported by evidences found in their orthologous protein annotations. Orthologous proteins are generally believed to have similar functions, and the orthologs can be obtained from Inparanoid [31]. For example, the *H. sapiens* gene POLA2, DNA polymerase subunit alpha B [Swiss-Prot: Q14181], was predicted by CSIDOP to exhibit alpha DNA polymerase activity (GO:0003889). Orthologs of POLA2 found by Inparanoid include: POL12 [ORF: YBL035C; SGD:S000000131] in *S. cerevisiae*, POLA2 [RGD:621817] in *R. norvegicus*, and CG5923 [FlyBase: FBgn0005696] in *D. melanogaster*. All three orthologs were associated with the alpha DNA polymerase activity (GO:0003889).

Furthermore, the CSIDOP method detected three molecular function terms for the human protein SLY, SH3 protein expressed in lymphocytes homolog [Swiss-Prot: O75995], while no information was found anywhere else. The three functions identified were DNA binding (GO:0003677), chromatin binding (GO:0003682), and zinc ion binding (GO:0008270). The SLY protein contains a COR1 chromatin-binding domain, and it was suggested in [38] that SLY may be targeted to the gonosomes in spermatids and may regulate gonosomal chromatin conformation and expression. Another protein CCNB3 [Swiss-Prot: Q8WWL7] in the human genome was predicted by the CSIDOP method to be involved in cyclin-dependent protein kinase regulator activity (GO:0016538) and protein binding (GO:0005515). An orthologous protein found in *D. melanogaster* CG5814 [FlyBase: FBgn0015625] shared both functional annotations, which were inferred from sequence or structural similarity and physical interaction [39], respectively. In the literature, CCNB3 was described as sharing properties with both A- and B-type cyclins. Cyclins play a key role in controlling progression through the cell cycle. They act as regulatory subunits of p34cdc2/CD28 and related cyclin-dependent protein kinases (cdks) [40]. In [41], CCNB3 was found to interact with the cyclin-dependent kinase CDK2, which implies that it indeed participates in protein binding and cyclin-dependent protein kinase regulator activity. Some of the 181 novel functional annotations found with supporting evidences can be found in supplementary Table S1. A complete list of the novel predictions can also be found in supplementary material (Text S3).

## Discussion

The CSIDOP is shown above to produce highly accurate function predictions for proteins in *H. sapiens*. To demonstrate its robustness, we further analyzed the method for its performance on *D. melanogaster*. For this study, we integrated protein interaction data from *S. cerevisiae*, *C. elegans*, and *H. sapiens* to form the reference dataset to determine functional annotations of proteins in *D. melanogaster*, the target dataset. None of the protein pairs in *D. melanogaster* were involved in training our model. In other words, the interacting domain patterns were extracted purely based on interaction pairs from *S. cerevisiae*, *C. elegans*, and *H. sapiens*. Function annotations were effectively assigned for 447 *D. melanogaster* proteins. Among the 447 proteins, CSIDOP accurately assigned function annotations to 419 proteins (i.e. 93.73% in accuracy).

In addition, we were able to discover novel annotations for some proteins. For example, the *D. melanogaster* protein CG15912 [Swiss-Prot: Q9W4J7] was detected by CSIDOP to exhibit ATPase activity, coupled to transmembrane movement of ions, phosphorylative mechanism (GO:0015662). Its orthologs: Haloacid dehalogenase-like hydrolase domain containing 3 [Swiss-Prot: Q9BSH5] in *H. sapiens* and [Swiss-Prot: Q9CYW4] in *M. musculus* were both found to be associated with phosphoglycolate phosphatase activity (GO:0008967) and hydrolase activity (GO:0016787), which is an ancestor term of our predicted term (GO:0015662). Moreover, for the protein CG18445 [Swiss-Prot: Q9V5F2], a multispan transmembrane protein related to fly Porcupine, our algorithm identified to carry out the O-acyltransferase activity (GO:0008374). Through literature search, we discovered that biological experiments conducted by Kraut et al. [42] confirmed the findings for CG18445.

Since the CSIDOP method only keeps the most significant interacting domain patterns from the closely related protein interaction pairs across species, PPI pairs in the test dataset not containing the patterns in the lookup table will result in no prediction. To enlarge the coverage, we can use a two-step prediction method: the first step will predict functions for a large number of proteins with lower confidence, and the second step uses CSIDOP for more accurate prediction. In the first step, for each protein pair in the test dataset, we construct a list of all interacting domain patterns. Then for each of these plausible domain patterns, we try to collect a list of protein interaction pairs in the reference dataset that contain the pattern. Numerous interaction pairs with shared pattern may exist in the reference dataset, and certain functions annotated to those pairs may be more likely to be associated with the target protein pair than other functions. Thus, in order to assess the probability of each functional assignment, we calculate the conditional probability of a protein interaction pair having function pair *F_1_*–*F_2_* given interacting domain pattern *D_1_*–*D_2_* (Eq. 1), where ‘-’ denotes interaction. In other words, *F_1_* and *F_2_* represent function assignments to proteins in the query interaction pair with modular domains *D_1_* and *D_2_*, respectively.
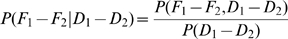
(1)where P(*F_1_*–*F_2_*, *D_1_*–*D_2_*) is calculated by counting the number of interaction pairs in the reference dataset that contain the interacting domain pattern *D_1_*–*D_2_* and have the corresponding functional annotation of *F_1_*–*F_2_*, and P(*D_1_*–*D_2_*) is computed by counting the number of pairs that contain the interacting domain pattern *D_1_*–*D_2_*. For a query protein interaction pair, the posterior probabilities of all possible function pairs are calculated, and finally, the top ranking function pairs are assigned. In this step, we were able to predict function assignments for 1546 human proteins, but with lower accuracy of 90%.

**Figure 3:**
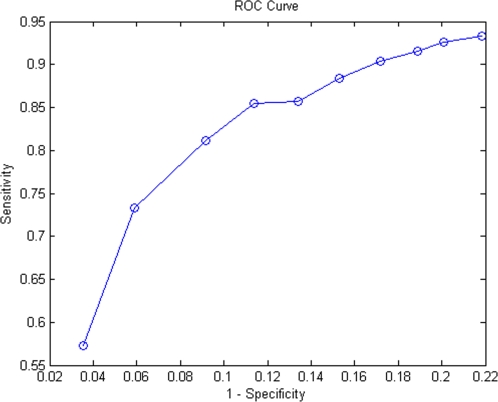
ROC curve. Sensitivity = TP/(TP+FN) Specificity = TN/(TN+FP) Function terms with probability above certain threshold are considered to be positive predictions and terms below the specified threshold are treated as negative predictions. The observed positive set of *g-t* association is obtained from the GO. The negative association set is defined as follows: if the association is not found in the positive set and term *t* is neither ancestor nor descendant of the known function terms in GO hierarchy for gene *g*. Therefore, true positives (TP) in this case refer to the overlaps between our positive predictions and observed positive set. True negatives (TN) are the overlaps between our negative predictions and the observed negative set. False positives describe *g-t* associations exist in our positive prediction list, but should be in the negative set. False negatives are *g-t* associations in our negative prediction list, but should be in the positive list.

Since prediction in the first step is based on probability of a protein *p* having term *t*, terms with probabilities above certain threshold can be treated as positive prediction and terms below the specified threshold can be treated as the negative prediction; thus, sensitivity and specificity measures can be calculated. Applying the same criteria in Nariai et al. [13], where they defined sensitivity as TP/(TP+FN), which corresponds to recall, and defined specificity as TN/(TN+FP), which corresponds to precision. A set of observed positive *p-t* association is obtained from the GO. The observed negative association set is defined as follows: if the association is not found in the positive set and term *t* is neither ancestor nor descendant of the known function terms in GO hierarchy for protein *p* [13]. Intuitively, true positives (TP) in this case refer to the overlaps between our positive predictions and the observed positive set, and true negatives (TN) are the overlaps between our negative predictions and the observed negative set. False positives are the *p-t* associations in our positive prediction list, but are observed to be in the negative set by GO. Lastly, false negatives are the *p-t* associations in our negative prediction list, but should be in the positive list. For varying posterior probability cutoffs, the relationship between sensitivity and 1-specificity is plotted in a ROC curve (Fig. 3). It is shown that the specificity of 96% with a sensitivity of 57% was achieved. When the specificity was lowered to 78%, the sensitivity increased dramatically to 93%.

### Conclusion

In this research, we describe CSIDOP, a novel approach to the protein function detection problem by extracting the conserved interacting domain patterns from protein interaction pairs across organisms. The CSIDOP method is assessed, both biologically and statistically, on the *Homo sapiens* genome for function annotation based on domain patterns extracted from interacting protein pairs in *S. cerevisiae*, *C. elegans*, *D. melanogaster* and *H. sapiens*. It makes functional assignments from a pool of 2,972 unique functional categories. The number of unique terms is considerably larger than the number of categories utilized in previous attempts. Using the *H. sapiens* genome, the CSIDOP method accurately assigned functions to 95.42% of the proteins when 2,972 function terms were used, which is highly reliable and is of practical use. The accuracy increased to 98.85% when the number of terms was decreased to 129. In contrast, with the same testing dataset, the Majority Rule algorithm, the simple domain based method, and orthology based method achieved only 59.50%, 61.98%, and 83.86% in accuracy, respectively. In this paper, we have shown that the CSIDOP method can not only provide additional functions to the incomplete GO annotations, but also assign functions for 181 human proteins that currently do not have GO functional terms. Supporting evidences for several of these newly annotated proteins can be found from other data sources or biological experiments, confirming the utility of this approach.

As more genomes are sequenced, there will be a growing need for better functional annotation of these genomes. In this paper, we have shown that an *in silico* method based on protein-protein interaction data and common domain interaction patterns is reliable for large-scale protein function discovery. Certainly, the CSIDOP method is not perfect, and it is limited in predicting functions for proteins with a priori knowledge of interactions. It cannot make predictions if the domain interaction patterns are not found in the lookup table. This method will continue to improve as protein-protein interaction data are increased in quality and quantity, and will readily scale to a genome-wide application.

## Materials and Methods

### Data sources

Protein interaction pairs were collected from the DIP, BioGRID, and MINT databases [43]–[45] for the organisms *S. cerevisiae*, *C. elegans*, and *D. melanogaster*. The human protein interaction data were obtained from the HPRD database [46]. Since we are concentrating on protein-protein interaction pairs with similar interacting domain patterns, proteins with no domain information were excluded. In addition, for the purpose of training our model, the training dataset does not contain any uncharacterized proteins. After data processing, the final training datasets consist of 11151, 231, 7709, and 13596 interaction pairs from *S. cerevisiae*, *C. elegans*, *D. melanogaster*, and *H. sapiens*, respectively. The CSIDOP method performance is assessed using the test dataset of 3812 human interaction pairs. The human training and test datasets do not contain any common protein interaction pairs.

**Figure 4:**
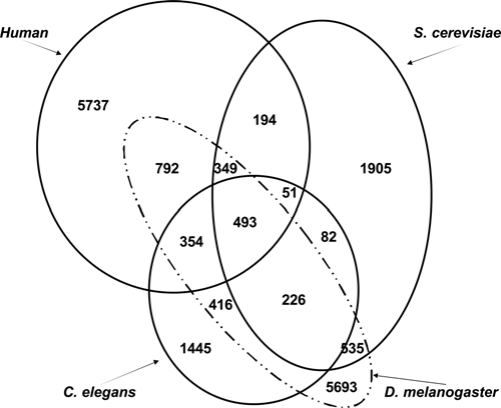
Domain distribution of organisms: *S. cerevisiae, C. elegans, D. melanogaster*, and *H. sapiens.* In our interaction data, the four organisms share 493 domains in common as shown in the figure. There are total 1603, 1489 and 1988 common domains between *D. melanogaster* and the other three organisms, *S. cerevisiae*, *C. elegans*, and Human, respectively.

Protein domain information was extracted from PFAM [29]. For each protein, Pfam-A and Pfam-B domains were considered. Among our interaction datasets, there are 3835, 3209, 8858, and 8112 unique domains in *S. cerevisiae*, *C. elegans*, *D. melanogaster*, and *H. sapiens*, respectively. There are a total of 493 unique Pfam domains in common between the four species. Complete information regarding domain distribution across the four organisms is shown in Figure 4. The protein ‘molecular function’ annotations were obtained from the Gene Ontology (GO) February, 2006 release [3]. Within the dataset, there are total 2,972 unique GO annotated molecular function terms.

### The CSIDOP method

The basic idea of the CSIDOP method is to assign appropriate GO functional annotations to proteins according to the interaction pairs in diverse species having the shared domain patterns. Domain patterns have been successfully applied in prediction of protein-protein interactions (PPIs) [47]–[51], a problem related to but different from protein function predictions. In protein interaction prediction, it mainly focuses on identifying interacting domains. While in our case, we aim to find modular domains that likely possess certain functions.

**Figure 5:**
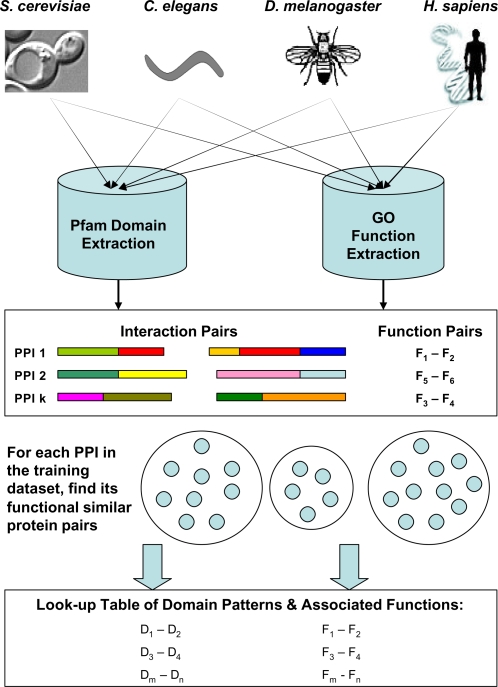
Flowchart of the CSIDOP method. The model begins with a collection of protein interaction pairs across various species and their domain and function information. For each PPI pair in the training dataset, we try to find its functional similar neighbors and form a group. Then from this group of PPIs with similar functions, we derive significant interacting domain patterns. This process is performed over all PPIs in the training dataset and in turn builds up a lookup table of patterns and associated functional assignments.

In order to extract the true functional interacting domain patterns from the vast wealth of deposited protein-protein interaction (PPI) data, we have devised an algorithm to find groups of protein interaction pairs with similar functions and applied χ^2^ statistics to derive meaningful interacting domain patterns from these PPI groups. Figure 5 shows the flowchart of the CSIDOP approach. For each protein interaction pair in the reference dataset, we tried to identify its neighbors based on functional distances between their individual proteins. In an earlier work by Resnik [52], functional similarity between two GO terms is measured based on their distances to the closest common ancestor term. Later on, Schlicker et al. [53] introduced a new measure that takes into account how detailed the lowest common ancestor is. Most recently, Wang et al. [54] measured the GO terms similarity by considering not only the number of but also the locations of common ancestor terms. In our study, we employed a slightly different definition of functional similarity between terms. Since the GO database is designed as a directed acyclic graph where each node represents a GO term, distance between two proteins can be defined as the closest GO-graph-node distance between all of their annotated molecular function terms. The GO-graph-node distance is described as the number of nodes separating two GO function terms in the graph.

In the training phase, for each PPI pair in the reference dataset, we tried to determine its close neighbors or functional similar interaction pairs. More precisely, each PPI pair in the training dataset serves as a centroid to form a group of protein pairs with similar functions. In doing so, all remaining pairs are compared against this centroid interaction pair. An incoming PPI pair is accepted to join the group if and only if the distances among individual proteins in the centroid pair and the pair under consideration are below certain threshold *t*. For instance, assume that there are two PPI pairs A–B and C–D, where ‘-’ denotes interaction. The two pairs are grouped together if and only if the following condition is satisfied; the closest GO-graph-node distance between either (A, C) and (B, D) or (A, D) and (B, C) are less than or equal to the threshold. In the end, PPI pairs in the same group are assuredly more likely to share the same or similar functions. In our application, the value of *t* is empirically set to be two.

After constructing a group of functional similar PPI pairs, we derive the most representative interacting domain patterns from each. This is accomplished by identifying an interaction domain pattern that is uniquely conserved in a group of PPI pairs across different organisms and with the same or similar functions (i.e. in the same group). Proteins often contain multiple domains, and one or more domains may form a functional unit during interaction, which we call a modular domain. Thus different combinations of modular domains in a protein should be considered in generating the potential interacting domain patterns. Due to the existence of some big proteins with more than 15 domains, it is computationally intensive and impractical to generate all possible combinations; therefore, measures had to be taken to trim down the set of all possible combinations by restricting the domain size of each protein to 4. While domain combinations involving more domains from each protein could slightly increase the prediction accuracy, they require much longer computational time. The assumption is also biologically reasonable because it is unlikely for a large number of domains to come together and form a single unit during interaction. Moreover, the same set of a large number of domains is unlikely to occur repeatedly in other proteins.

In consequence, a list of potential interacting domain patterns is enumerated from each protein pairs in an individual group of PPIs with similar functions. Each domain pattern will be associated with a list of function terms from their corresponding PPI pairs. In order to select the most significant interacting domain patterns, χ^2^ statistics is calculated for each pattern. The χ^2^ value is computed using the following formula,
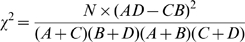
(2)
*N* is the total number of PPI pairs in the reference dataset. Variable *A* is the number of PPI pairs in the group that contain the particular ‘pattern’, and *B* is the number of remaining PPI pairs outside the group that contain the ‘pattern’. Variables *C* and *D* are the number of PPI pairs that do not contain the ‘pattern’ in the group and in the remaining samples outside the group, respectively. An interacting domain pattern occurring more frequently in PPI pairs inside the group than outside the group is expected to have a higher χ^2^ value, hence is more significant. Finally, the deduced interacting domain patterns with the highest χ^2^ value are adopted in a lookup table for function annotation.

## Supporting Information

Text S1 A list of domains and their corresponding IDs
(0.29 MB TXT)

Text S2 A lookup table of domain patterns and associated functional assignments
(0.62 MB TXT)

Text S3 A complete list of novel functional predictions for proteins in *H. sapiens* in text format
(0.01 MB TXT)

Table S1 Novel functional annotations for some *H.sapiens* proteins found with supporting evidences. For each human protein in the 1st column, highlighted terms in the 2nd column are the GO terms that CSIDOP predicted and also supported by evidence found in other databases or literature. The evidence is shown in the 3rd column where it lists the orthologous or paralogous proteins annotated with these highlighted terms inferred using different techniques. For example, we predicted the protein Q96A23 to have the function GO:0001786, and we found that its paralog Q99829 protein in *H. sapiens* is detected with GO:0001786 through the evidence code IDA. InterPro is a database of protein families, domains and functional sites in which identifiable features found in known proteins can be applied to unknown protein sequences. IntAct is by Giot et al. The following is a list of evidence codes used in the table.
(0.04 MB DOC)
