# Supplementary material for: Protein Function Assignment through Mining Cross-Species Protein-Protein Interactions
Source: PLoS One. 2008 Feb 6;3(2):e1562. doi: 10.1371/journal.pone.0001562 (PMC2216687; doi:10.1371/journal.pone.0001562)
Supplement: Table S1 — Novel functional annotations for some H.sapiens proteins found with supporting evidences. For each human protein in the 1st column, highlighted terms in the 2nd column are the GO terms that CSIDOP predicted and also supported by evidence found in other databases or literature. The evidence is shown in the 3rd column where it lists the orthologous or paralogous proteins annotated with these highlighted terms inferred using different techniques. For example, we predicted the protein Q96A23 to have the function GO:0001786, and we found that its paralog Q99829 protein in H. sapiens is detected with GO:0001786 through the evidence code IDA. InterPro is a database of protein families, domains and functional sites in which identifiable features found in known proteins can be applied to unknown protein sequences. IntAct is by Giot et al. The following is a list of evidence codes used in the table. (0.04 MB DOC) [file pone.0001562.s004.doc]

**Table S1.** Novel functional annotations for some *H.sapiens* proteins found with supporting

evidences

| **Human Proteins in**  **Swiss-Prot ID** | **Predicted Functions** | **Verification with Evidence** |
| --- | --- | --- |
| Q96A23 – Copine-4 (CPNE4) | **GO:0001786** – Phosphatidylserine binding  **GO:0005215** – Transporter activity  **GO:0005544** – Calcium-dependent phospholipid binding | GO:0001786 – Q99829 in *Hsa* (IDA)  GO:0005215 – Q9UBL6, O95741, Q99829 in *Hsa* (TAS)  GO:0005544 – At5g61900 in *Ath* (IDA), At5g07300 & At1g08860 in *Ath* (NAS), At5g61900 in *Ath* (RCA), Q99829 in *Hsa* (TAS) |
| O00167 – Eyes absent homolog 2 (EYA2) | **GO:0000287** – Magnesium ion binding  **GO:0004725** – Protein tyrosine phosphatase activity  **GO:0016787** – Hydrolase activity  GO:0031177 – Phosphopantetheine binding | GO:0000287, GO:0004725, and GO:0016787 are also detected by QuickGO with UniProt Keyword (IEA).  GO:0004725 was also detected for ortholog MGI:109341 in *Mmu* (IDA) and RGD:620096 in *Rno* (ISS). |
| P55287 – Cadherin-11 precursor (CDH11) | **GO:0005509** – Calcium ion binding  **GO:0015643** – Toxin binding  GO:0050839 – Cell adhesion molecule binding  **GO:0004872** – Receptor activity  **GO:0008013** – Beta-catenin binding  GO:0005524 – ATP binding  GO:0005198 – Structural molecule activity | Cadherins are calcium dependent cell adhesion proteins.  GO:0005509 – InterPro (IEA)  Paralogs Q86UP0 (IDA) & Q02413 (NAS) in *Hsa.* are also annotated with GO:0005509  FBgn0003391 in *Dme* is also annotated with GO:0004872 (ISS) and GO:0008013 (NAS)  GO:0015643 – Q02413 in *Hsa* has the annotation (NAS) |
| Q96FN4 – Copine-2 (CPNE2) | GO:0001786 – Phosphatidylserine binding  **GO:0005215** – Transporter activity  **GO:0005544** – Calcium-dependent phospholipid binding | GO:0005215 – MGI:1917818 in *Mmu* (ISS, RCA), RGD:1310178 in *Rno* (ISS), O75131, Q9UBL6, O95741 in *Hsa* (TAS)  GO:0005544 – At5g61900 in *Ath* (IDA, RCA), MGI:1917818 in *Mmu* (ISS, RCA), RGD:1310178 in *Rno* (ISS), At5g07300 & At1g08860 in *Ath* (NAS), O75131 in *Hsa* (TAS) |
| Q9H222 – ATP-binding cassette sub-family G member 5 (ABCG5) | **GO:0016887** – ATPase activity  **GO:0000166** – Nucleotide binding  **GO:0005524** – ATP binding  **GO:0042626** – ATPase activity, coupled to transmembrane movement of substances  GO:0005224 – ATP-binding and phosphorylation-dependent chloride channel activity  GO:0005260 – Channel-conductance-controlling ATPase activity | GO:0016887 – InterPro (IEA)  GO:0000166 – InterPro (IEA) & UniProt Keyword (IEA)  GO:0005524 – InterPro (IEA) & UniProt Keyword (IEA)  GO:0042626 – FBgn0039244 in *Dme*, YOL075C, S000000604 in *Sce* (ISS) |
| O75553 – Disabled homolog 1 (DAB1) | GO:0005068 – Transmembrane receptor protein tyrosine kinase adaptor protein activity  GO:0005158 – Insulin receptor binding  GO:0005159 – Insulin-like growth factor receptor binding  **GO:0005515** – Protein binding  **GO:0005543** – Phospholipid binding | GO:0005515 – MGI:108554 in *Mmu* (TAS)  GO:0005543 – MGI:108554 in *Mmu* (TAS) |
| P59780 – AP-3 complex subunit sigma-2 (AP3S2) | **GO:0005215** – Transporter activity | GO:0005215 – Q92572 in *Hsa* (NAS) |
| Q9UEU0 – Vesicle transport through interaction with t-SNAREs homolog 1B (VTI1B) | **GO:0005485** – v-SNARE activity | GO:0005485 – FBgn0035156 in *Dme* (ISS), SPBC3B9.10 in *Spo* (ISS), S000004810 in *Sce* (TAS) |
| Q8NES3 – Beta-1, 3-N-acetylglucosaminyltransferase lunatic fringe (LFNG) | **GO:0016757** – Transferase activity, transferring glycosyl groups  **GO:0008194** – UDP-glycosyltransferase activity  **GO:0008375** – Acetylglucosaminyltrasferase activity  GO:0005102 – Receptor binding | GO:0016757 – InterPro & UniProt Keyword (IEA) and RGD:620587 in *Rno* (ISS)  GO:0008194 – FBgn0011591 in *Dme* (IDA)  GO:0008375 – FBgn0011591 in *Dme* (IDA, NAS, TAS) |
| Q13439 – Golgin subfamily A member 4 (GOLGA4) | **GO:0008017** – Microtubule binding  **GO:0005200** – Structural constituent of cytoskeleton  GO:0005088 – Ras guanyl-nucleotide exchange factor activity | GO:0008017 – FBgn0029688 in *Dme* (IDA)  GO:0005200 – FBgn0013988 in *Dme* (ISS) |

For each human protein in the 1^st^ column, highlighted terms in the 2^nd^ column are the GO terms that CSIDOP predicted and also supported by evidence found in other databases or literature. The evidence is shown in the 3^rd^ column where it lists the orthologous or paralogous proteins annotated with these highlighted terms inferred using different techniques. For example, we predicted the protein Q96A23 to have the function GO:0001786, and we found that its paralog Q99829 protein in *H. sapiens* is detected with GO:0001786 through the evidence code IDA. InterPro is a database of protein families, domains and functional sites in which identifiable features found in known proteins can be applied to unknown protein sequences. IntAct is by Giot et al. The following is a list of evidence codes used in the table.

IEA = Inferred by Electronic Annotation IDA = Inferred from Direct Assay

ISS = Inferred from Sequence Similarity NAS = Non-traceable Author Statement

TAS = Traceable Author Statement RCA = Reviewed Computational Analysis

Keyword = Mappings inferred from UniProt keyword

The following lists abbreviations used for different organisms.

*Ath* = *A. thaliana Cel* = *C. elegans* *Dme* = *D. melanogaster*

*Rno* = *R. norvegicus* *Sce* = *S. cerevisiae* *Spo* = *S. pombe*
